# Light-Induced Fluorescence-Based Device and Hybrid Mobile App for Oral Hygiene Management at Home: Development and Usability Study

**DOI:** 10.2196/17881

**Published:** 2020-10-16

**Authors:** Jun-Min Kim, Woo Ram Lee, Jun-Ho Kim, Jong-Mo Seo, Changkyun Im

**Affiliations:** 1 Department of Electrical and Computer Engineering Seoul National University Seoul Republic of Korea; 2 Department of Electronics and Information Engineering Hansung University Seoul Republic of Korea; 3 Department of Electronic Communication Engineering Gyeonggi University of Science Technology Siheung Republic of Korea; 4 Department of Electrical Energy Engineering Keimyung University Daegu Republic of Korea; 5 Dental Research Institute Seoul National University Seoul Republic of Korea; 6 Dental Life Science Research Institute Seoul National University Dental Hospital Seoul Republic of Korea

**Keywords:** dental plaque, oral hygiene, red fluorescence, mobile health, deep learning, object detection, instance segmentation

## Abstract

**Background:**

Dental diseases can be prevented through the management of dental plaques. Dental plaque can be identified using the light-induced fluorescence (LIF) technique that emits light at 405 nm. The LIF technique is more convenient than the commercial technique using a disclosing agent, but the result may vary for each individual as it still requires visual identification.

**Objective:**

The objective of this study is to introduce and validate a deep learning–based oral hygiene monitoring system that makes it easy to identify dental plaques at home.

**Methods:**

We developed a LIF-based system consisting of a device that can visually identify dental plaques and a mobile app that displays the location and area of dental plaques on oral images. The mobile app is programmed to automatically determine the location and distribution of dental plaques using a deep learning–based algorithm and present the results to the user as time series data. The mobile app is also built with convergence of naive and web applications so that the algorithm is executed on a cloud server to efficiently distribute computing resources.

**Results:**

The location and distribution of users’ dental plaques could be identified via the hand-held LIF device or mobile app. The color correction filter in the device was developed using a color mixing technique. The mobile app was built as a hybrid app combining the functionalities of a native application and a web application. Through the scrollable WebView on the mobile app, changes in the time series of dental plaque could be confirmed. The algorithm for dental plaque detection was implemented to run on Amazon Web Services for object detection by single shot multibox detector and instance segmentation by Mask region-based convolutional neural network.

**Conclusions:**

This paper shows that the system can be used as a home oral care product for timely identification and management of dental plaques. In the future, it is expected that these products will significantly reduce the social costs associated with dental diseases.

## Introduction

Dental plaque is a sticky biofilm associated with oral diseases such as tooth decay and periodontal disease. Management of dental plaque is one of the effective ways to prevent dental diseases, but its transparent and colorless properties make it difficult to visually identify and manage. Therefore, it is important to improve motivation for dental plaque management by making it easy to identify whether the dental plaque adheres to the tooth surface. The most commonly recommended method is to use a disclosing agent that can identify the dental plaque by staining [1,2]. However, the assessment of dental plaque accumulation is subjective and error-prone because it is generally performed through self-monitoring. Therefore, accurate evaluation requires clinical examination by a clinician, which increases cost and time [3,4]. In addition, the mouth and tongue may be stained all day or temporarily by disclosing agents, and some products contain dyes that may cause allergic reactions [5].

It has been demonstrated that dental plaque can also be identified by the light-induced fluorescence (LIF) technique [6,7]. This technique is based on the red fluorescence property of porphyrins, metabolites of heterogeneous bacteria within dental plaque, when irradiated with narrow blue-violet light (centered at 405 nm wavelength) [8,9].

Since it was first reported in the 1920s that dental plaque emits red fluorescence by ultraviolet ray irradiation [10,11], various studies using LIF characteristics have been published, such as fluorescence changes according to oral bacterial species, fluorescence imaging systems, and clinical diagnosis. Studies of fluorescence changes by oral bacterial species reported that red fluorescence was detected in *Prevotella intermedia*, *P. melaninogenica*, *Actinomyces naeslundi*, *A. israelii*, and *Bifidobacterium dentium*, green fluorescence was observed in *Streptococcus oralis*, *S. salivarius*, *S. mutans*, *S. mitis*, *S. sobrinus*, *Fusobacterium nucleatum*, and *Propionibacterium acnes*, and orange fluorescence was found in *Latobacillus fermentans*, *L. rhamnosus*, and *L. casei*, and *Candida albicans* [8,12-14]. Commercial products reported in the scientific literature as LIF imaging systems for dental plaque detection include ACTEON SOPROLIFE (Henry Schein, Inc) [15] and QLF-D Biluminator (Inspektor Research Systems BV) [16]. These products have been used to evaluate plaque levels by comparing clinical and red fluorescent plaques [16-18], and some studies have shown that tooth defects such as caries, calculus, hypomineralization, and discoloration can be observed with red fluorescence [15,19,20]. However, these products are not only classified as medical devices, but also have problems in size and price, making them unsuitable for ordinary users to motivate plaque management at home.

Therefore, we judged that an inexpensive, compact, and intuitively usable system would be suitable for motivating plaque management to ordinary users and developed an oral hygiene monitoring system consisting of a LIF device and a smartphone-based mobile app. The LIF device induces the fluorescence of plaque and the app serves to visualize the plaque area, but in order to greatly motivate, an image processing method that emphasizes the plaque area is required. The traditional image processing method is sufficient to emphasize the plaque area in a limited environment, but an ordinary user needs an image processing method suitable for taking oral images with various cameras such as an Android phone and an iPhone in various environments such as a room, a bathroom, and a living room. Recently, deep learning algorithms have been applied to detection, segmentation, classification, and prediction in various medical fields, including plaque classification, showing surprising results [21,22]. Therefore, it was determined that the deep learning algorithm would be the best way to solve the above-mentioned disadvantages.

In this paper, we introduce a deep learning–based oral hygiene monitoring system that makes it easy to identify dental plaques in our home. The system consists of a device that can visually identify dental plaques and a mobile app that displays the location and area of dental plaques on the oral image. The mobile app was developed based on two deep learning models to sequentially detect tooth areas and highlight plaque areas.

## Methods

### Overview

**Figure 1:**
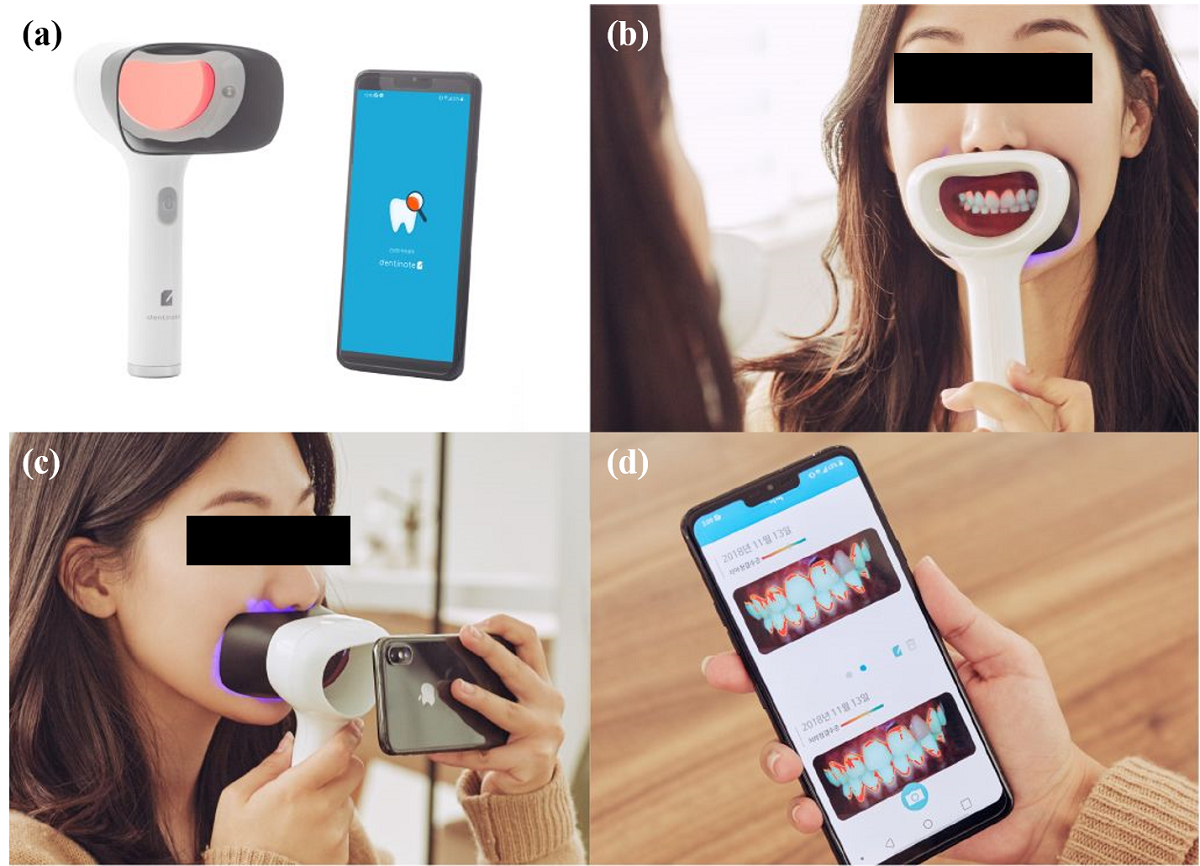
Oral hygiene monitoring system: (a) light-induced fluorescence device and hybrid mobile app; (b) method for monitoring oral hygiene with the naked eye through a mirror; (c) method for monitoring oral hygiene using smartphone; (d) hybrid mobile app that provides time series oral hygiene information.

In this study, we developed a hand-held LIF device that allows the user to easily monitor dental plaque with the naked eye in a mirror and a hybrid mobile app that provides oral hygiene information. Figure 1 shows the LIF device and the hybrid mobile app.

### Development of Light-Induced Fluorescence Hardware Interface

**Figure 2:**
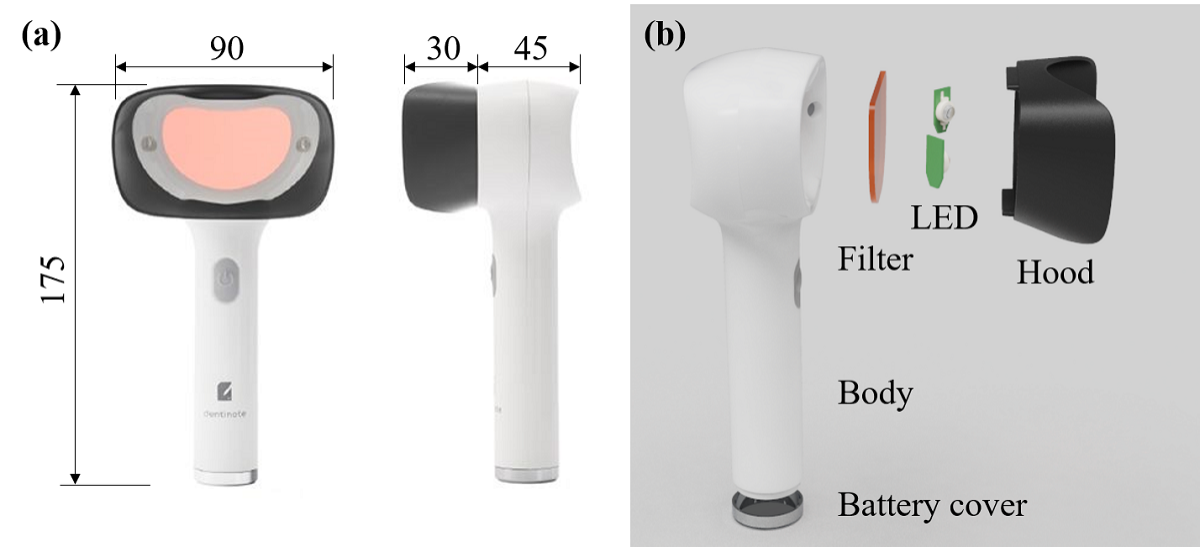
Light-induced fluorescence device for monitoring oral health: (a) dimensions (mm) and (b) components.

As shown in Figure 2, the LIF device consists of 5 parts: body, two light-emitting diodes (LEDs), filter, hood, and battery cover. The body has a cylindrical handle with 28 mm diameter for easy hand grip, a button manufactured through silicone insert injection molding for better assembly, and a wide viewer suitable for viewing the oral hygiene. The LED (GTPDTV64101, Shenzhen Getian Opto-Electronics Co Ltd) emits 1 W narrow-band spectrum in the wavelength range of 400 to 410 nm with the view angle of 120 degrees. The filter is manufactured through a color-mixing technique in injection molding so that it has a color correction function for identifying clean teeth as white and plaque as red in color. The hood is matte black to block ambient light and minimize light reflection and is interchangeable for each user. The battery cover is designed for high friction through serration technique and it is not easily separated from the body. The device is powered by three AAA batteries and can last for approximately two and a half hours.

### Development of Hybrid Mobile App

**Figure 3:**
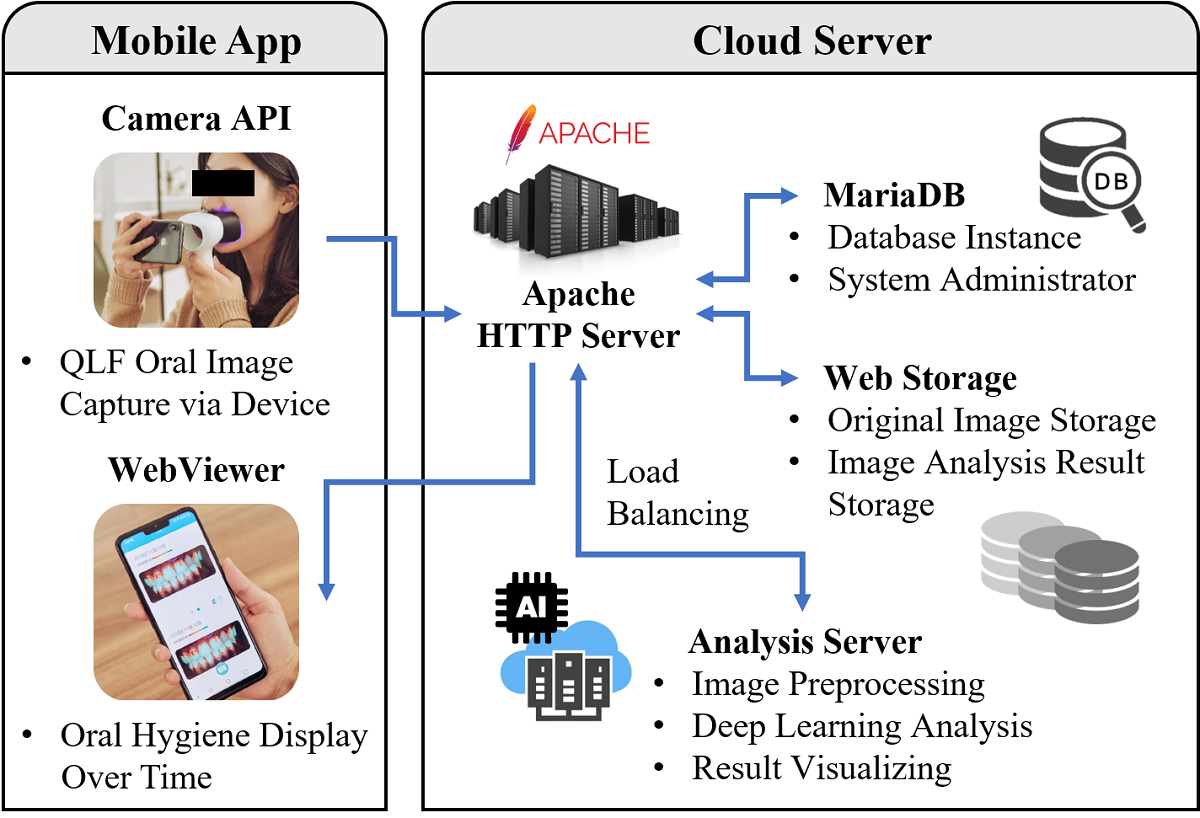
Architecture of the hybrid mobile app comprising mobile app and cloud server.

A hybrid mobile app is one that combines the functionalities of both a native app, which allows access to the smartphone camera, and web application, which can easily be updated without installation by modifying only the code on the server. In this study, we built a hybrid mobile app using JAVA for Android and Swift for iOS and, as shown in Figure 3, its architecture is divided into the mobile app part and the cloud server part. The mobile app consists of a camera application programming interface that captures LIF oral images via the device and a WebViewer that displays oral hygiene over time on the smartphone screen. The cloud server is built on Amazon Web Services, which has abundant computing resources such as CPU and GPU. It contains an Apache http server, which process requests and provides web assets and content over http protocol, a MariaDB, which transforms data into structured information, an Analysis server, which analyzes oral hygiene through deep learning algorithms, and web storage, which stores original images and analyzed results.

### Oral Image Data Flow on Cloud Server

An oral image taken with the user’s smartphone camera is uploaded to the http server implemented in Apache using the http protocol. The http servers use load balancing technology to send images to the deep learning–based analysis servers, reducing the load on analyzing large numbers of oral images from multiple users. After analysis is completed, the result image is converted into thumbnails to reduce the amount of transmission and reception and then sent to the user’s smartphone screen to check the oral hygiene information. Information such as account, date, plaque location, and cleanliness generated during the oral hygiene analysis are stored and updated in the database on the MariaDB as time series data.

### Deep Learning–Based Algorithm for Oral Hygiene Analysis

**Figure 4:**
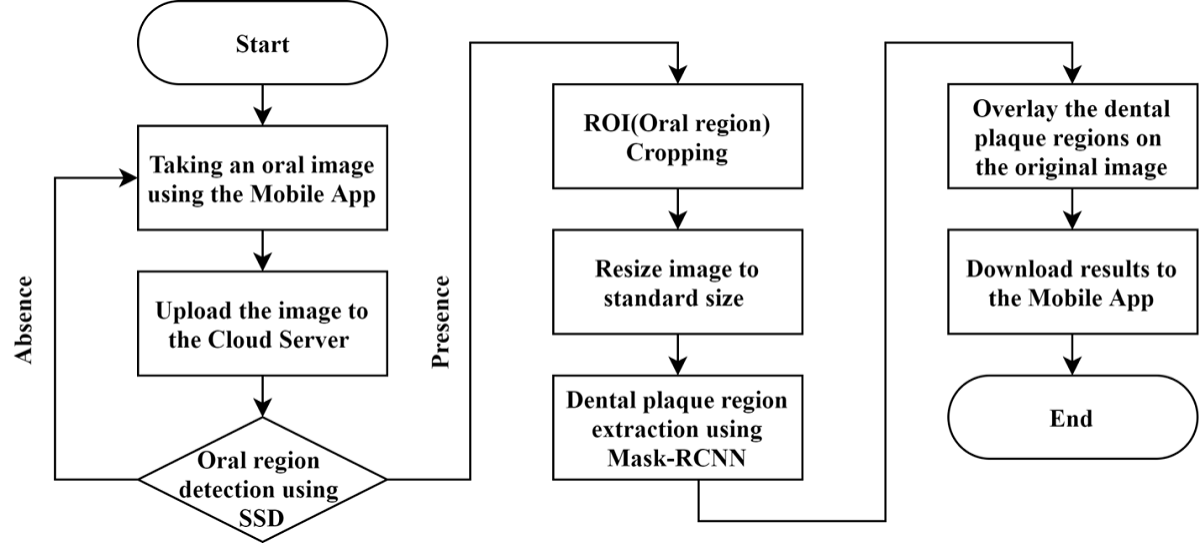
Flowchart of oral hygiene analysis.

The oral hygiene analysis is performed through deep learning–based image processing algorithms including object detection, which determines whether the input image is an oral image and localizes the oral region, and instance segmentation, which extracts the dental plaque regions. In this analysis, datasets categorized into oral and nonoral images are used. The 2000 oral images are taken only via the device and stored on the server, and the 2000 nonoral images are Pascal visual object classes images [23] without teeth or gums. Figure 4 illustrates a flowchart of the oral hygiene analysis.

For the object detection, the ground-truth bounding box annotations are first performed, which outline the region of interest (RoI) involving only the teeth and gums for each image. Then the single shot multibox detector (SSD) [24], one of the most popular deep learning models for object detection, is applied to detect the RoI within the images (predicted bounding box). The SSD model training is performed with a training dataset after dividing the datasets into a 2000-image training dataset (1000 oral and 1000 nonoral images) and a 2000-image test dataset (1000 oral and 1000 nonoral images). In order to improve the learning performance, the training dataset is augmented with random sample crop, photometric distortion (random transformations in the color HSV domain), rotation, and mirroring. Then the resolutions of the images in the dataset are all converted to 300×300 pixels, and their RGB values are normalized between 0 and 1.

In order to finely extract the red fluorescence–emitted dental plaque region from the oral image, an instance segmentation technique capable of classification and detection of multiple instances in one class is required.

In this study, the Mask region-based convolutional neural network (R-CNN) [25], which is a Faster R-CNN [26] with the addition of a small fully convolutional network that can act as object detection and mask segmentation for each RoI, is used as an instance segmentation technique. The RoI images extracted through the SSD model are used as the input data for Mask R-CNN model training.

Pixel-level annotation for Mask R-CNN training was performed on plaque areas emitting red fluorescence in tooth images according to three criteria:

1. Connected components labeling, which groups its pixels into components based on pixel connectivity, is performed on plaque areas
2. Plaque areas that span several teeth are divided at tooth boundary
3. Plaque thinly distributed between teeth is classified as one instance without following the second criterion

The images are technically prepared in the Common Objects in Context (COCO) data format [27] and augmented with rotation and aspect ratio conversion.

The Mask R-CNN model uses a model pretrained with COCO data as an initial parameter, and the training of the model is performed by the loss function–based stochastic gradient descent method.

This research protocol was approved by the institutional review board (IRB #ERI19046), Seoul National University Dental Hospital. In order to protect users’ privacy information, no personally identifiable information such as name, age, or gender are included in the image, and except for the mouth and the device, the visible parts of the image were mosaicized to make them indistinguishable.

## Results

### System Characterization

The LIF device consists of a body for easy grip, two LEDs for 400 to 410 nm light emission, filter for color correction, and hood for blocking ambient light, as shown in Figure 2. The electrical features of the device are power consumption of about 2.1 W, current of about 468 mA with 4.5 V, and idle current of about 4.5 mA. When using three AAA batteries with a capacity of 1500 mAh in series, continuous use time is about 150 minutes, and an individual can use it for about 75 days if it is used for 2 minutes per day.

**Figure 5:**
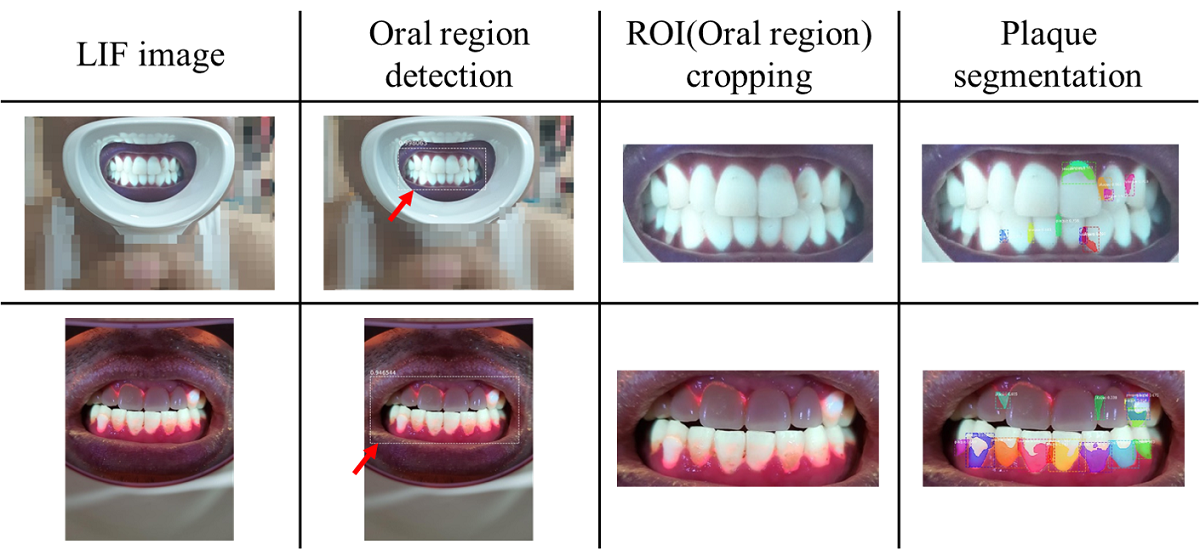
Representative images obtained during deep learning–based oral hygiene analysis. The red arrow points to the predicted bounding box.

The hybrid mobile app has a scrollable web view, allowing users to easily observe changes in dental hygiene status using time series data as shown in Figure 1(d). Oral hygiene analysis is then performed by taking an oral image through the app and sending it to the cloud server (Figure 3). After analysis, the oral hygiene results are sent back to the user’s app. It takes 3.00 (SD 0.020) seconds for the deep learning graphics processing unit and other libraries to load, 1.69 (SD 0.019) seconds for the oral region detection process (SSD), and 4.38 (SD 0.024) seconds for the dental plaque region extraction process (Mask R-CNN). Figure 5 presents representative results obtained during object detection and instance segmentation.

### Experimental Analysis of Plaque Detection Algorithm

Oral hygiene analysis was performed through object detection and instance segmentation, and each performance was verified using intersection over union (IoU) and average precision (AP). IoU is an evaluation metric used to verify the performance of object detection. Its value is calculated by dividing the area of overlap by the area of union of both the ground-truth bounding box and predicted bounding box. AP, also a metric for evaluating the accuracy of the object detector, is the average of precision values corresponding to recall values between 0 and 1. Here, recall indicates how well all positives are predicted, and precision indicates the accuracy of the predicted result. The object detection performance of SSD is expressed by AP, which is an average value for IoU threshold. The AP values at IoU thresholds of 0.50, 0.60, 0.70, 0.80, and 0.90 are 90.9, 81.8, 63.6, 42.0, and 9.1, respectively. The average over IoU thresholds from 0.50 to 0.95 with a step size of 0.05 is 53.31. The performance of Mask R-CNN is expressed as IoU for the whole segmentation result instead of AP because the dental plaque regions are atypical, and the result value is 0.31.

## Discussion

### Principal Findings

In this paper, we presented a deep learning–based oral hygiene monitoring system consisting of a LIF device and hybrid mobile app to facilitate oral hygiene at home using a smartphone. The most prominent feature of the LIF device is the filter. Filters of similar devices are manufactured by depositing a thin dielectric layer onto a glass substrate, while the filter of our device is simply manufactured by adopting a method of mixing colors with poly(methyl methacrylate). Thus, the filter of our device is relatively low in manufacturing cost while maintaining the main role of the filter to make the red fluorescence of the plaque stand out compared with the surrounding tooth color. However, performance of the filter is affected by the intensity of ambient light [28], so a hood is required to minimize degradation by ambient light. Although our device also has a black hood, it has been observed that overexposed images are taken because the hood cannot completely block ambient light outdoors or under strong lighting. These results are caused by light leakage due to different oral structures of individuals, and it is expected that these problems will be sufficiently solved when the hood is made of flexible materials or customized. Another source of ambient light is the large window for visual observation or camera shooting. This is fundamentally unable to block ambient light, but it is believed that providing a light-blocking agent will minimize the influence of ambient light.

The key feature of our hybrid mobile app is that it is programmed with a deep learning algorithm. Since most conventional LIF products are developed for medical purposes, nonmedical users require the help or education of a medical practitioner. On the other hand, our mobile app automatically determines the location and distribution of dental plaques without clinical examination or training. In addition, since the result is stored and displayed as time series data, it is convenient for the user to manage oral hygiene. The deep learning models for oral detection and dental plaque segmentation require high computing resources, but by performing analysis on the server, computing resources can be effectively managed, and results can be quickly generated.

**Figure 6:**
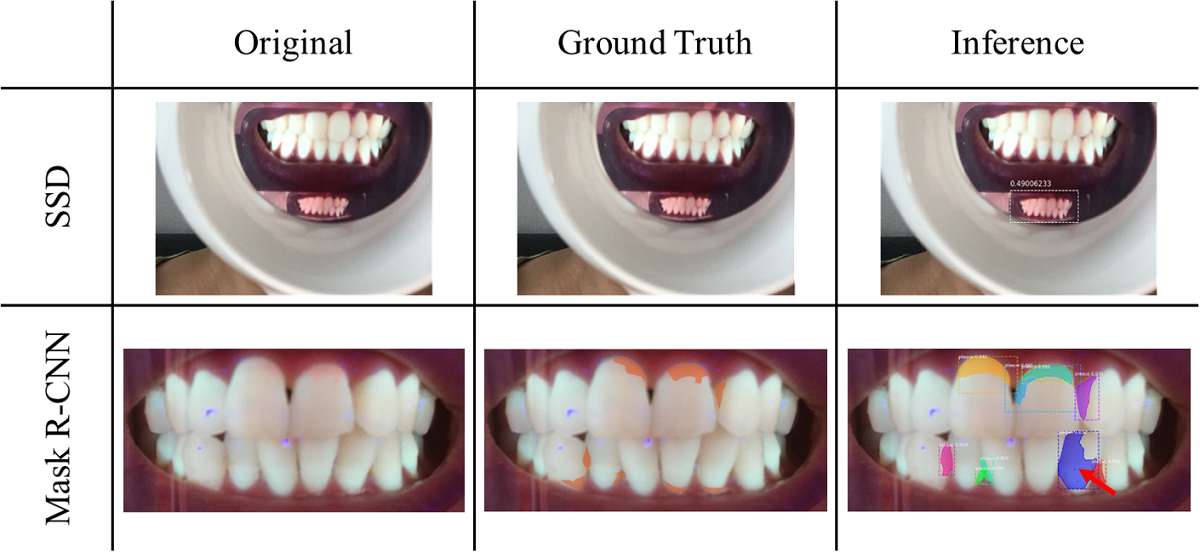
Representative poor results of each deep learning algorithm.

As shown in Figure 6, our segmentation algorithm is somewhat poor in performance. In order to determine the cause, 2000 images were randomly sampled from the images stored on the server and statistically analyzed. Images were categorized as normal, out-focusing, far away, overexposed, too dark, foggy, yawning, and no device. Each category was multiselected, and the results are shown in Table 1. Multimedia Appendix 1 shows representative images categorized in each category.

**Table 1:** Statistical results of the quality of the acquired images.

Category	Value n (%)
Normal	627 (31.4)
Out-focusing	882 (44.1)
Far away	501 (25.1)
Overexposed	489 (24.5)
Too dark	297 (14.9)
Foggy	249 (12.5)
Yawning	223 (11.2)

Of the images from the server, only 31.35% (627/2000) were classified as normal, meaning that most images were described with one or more variables such as out-focusing, far away, overexposed, too dark, foggy, and yawning, which is thought to directly affect the performance of deep learning–based analysis. In addition to these cases, two tooth areas were found by reflecting the tooth area on the smartphone case, and the plaque area was not properly divided when the captured image had a red tone. In the future, if we provide guidelines to users on an appropriate environment for acquiring images and improve the algorithms based on continuously increasing numbers of images as the number of users of the product increases, we expect that plaque identification will improve [29].

### Limitations

Due to the different image sensor characteristics of smartphones, a consistent preprocessing method could not be applied. The other limitation was that the size of the training data was smaller than the collected data due to various optical environments.

### Conclusion

The primary cause of dental caries and periodontal disease is the failure to remove dental plaque in a timely manner. Providing preventive care solutions to quickly identify and respond to dental plaques at home can significantly reduce social costs associated with oral disease. The LIF system introduced in this paper consists of a LIF device for visually identifying dental plaques and a mobile app for providing deep learning–based oral hygiene analysis results. The device allows the user to visually check oral hygiene in a mirror and the app motivates the user to perform oral hygiene management by providing the oral hygiene analysis results in time series. In this paper, we introduced a home oral care system, but in the future, we will introduce LIF-based medical devices for marginalized populations, including the elderly, people of lower socioeconomic standing, and those living where the internet is unavailable, by applying edge computing technique and developing low-cost devices in the form of smartphone accessories.

## Appendix

Multimedia Appendix 1 Representative images categorized into each category: (a) normal, (b) out-focusing, (c) far away, (d) overexposured, (e) foggy, and (f) yawning.

## Abbreviations

AP: average precision
COCO: Common Objects in Context
IoU: intersection over union
LED: light-emitting diode
LIF: light-induced fluorescence
R-CNN: region-based convolutional neural network
RoI: region of interest
SSD: single shot multibox detector
