# Supplementary figures and images for: Light-Induced Fluorescence-Based Device and Hybrid Mobile App for Oral Hygiene Management at Home: Development and Usability Study
Source: JMIR Mhealth Uhealth. 2020 Oct 16;8(10):e17881. doi: 10.2196/17881 (PMC7600004; doi:10.2196/17881)

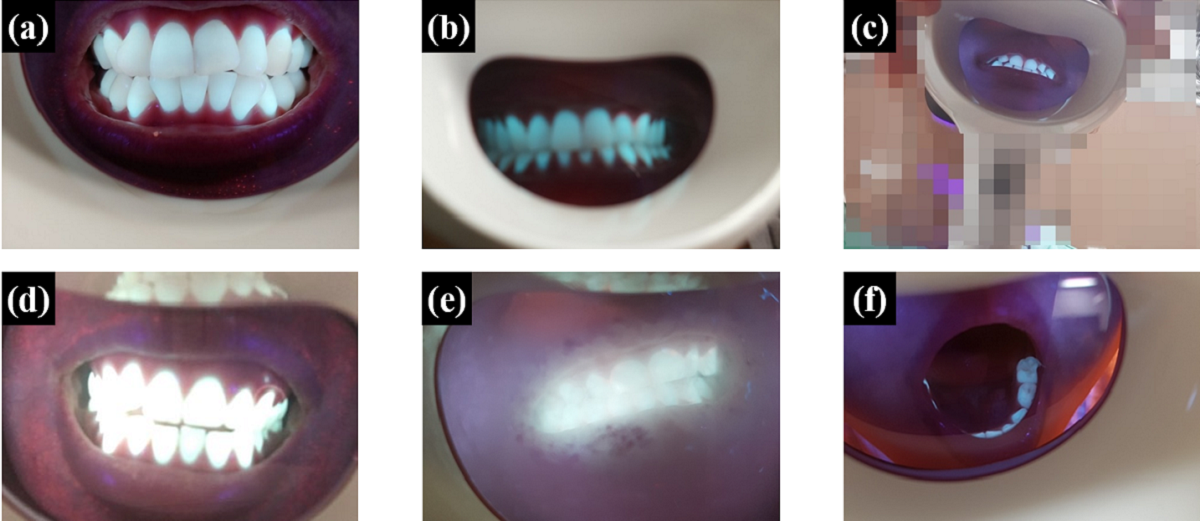

Supplement: Multimedia Appendix 1 [file mhealth_v8i10e17881_app1.png]
